# Imaging Artifacts of Nonadhesive Liquid Embolic Agents in Conventional and Cone-beam CT in a Novel in Vitro AVM Model

**DOI:** 10.1007/s00062-021-01013-5

**Published:** 2021-04-14

**Authors:** Niclas Schmitt, Ralf O Floca, Daniel Paech, Rami A El Shafie, Ulf Neuberger, Martin Bendszus, Markus A Möhlenbruch, Dominik F Vollherbst

**Affiliations:** 1 Department of Neuroradiology, INF 400, Heidelberg University Hospital, 69120 Heidelberg, Germany; 2 Medical Image Computing, German Cancer Research Center (DKFZ), Heidelberg, Germany; 3 Department of Radiation Oncology, Heidelberg University Hospital, Heidelberg, Germany; 4 Heidelberg Institute for Radiation Oncology (HIRO), National Center for Radiation Research in Oncology (NCRO), Heidelberg, Germany; 5 Department of Radiology, German Cancer Research Center (DKFZ), Heidelberg, Germany

**Keywords:** Arteriovenous malformation, Computed tomography, Onyx, EVOH, Embolization

## Abstract

**Background:**

A major drawback of liquid embolic agents (LEAs) is the generation of imaging artifacts (IA), which may represent a crucial obstacle for the detection of periprocedural hemorrhage or subsequent radiosurgery of cerebral arteriovenous malformations (AVMs). This study aimed to compare the IAs of Onyx, Squid and PHIL in a novel three-dimensional in vitro AVM model in conventional computed tomography (CT) and cone-beam CT (CBCT).

**Methods:**

Tubes with different diameters were configured in a container resembling an AVM with an artificial nidus at its center. Subsequently, the AVM models were filled with Onyx 18, Squid 18, PHIL 25% or saline and inserted into an imaging phantom (*n* = 10/LEA). Afterwards CT and CBCT scans were acquired. The degree of IAs was graded quantitatively (Hounsfield units in a defined region of interest) and qualitatively (feasibility of defining the nidus)—Onyx vs. Squid vs. PHIL vs. saline, respectively.

**Results:**

Quantitative density evaluation demonstrated more artifacts for Onyx compared to Squid and PHIL, e.g. 48.15 ± 14.32 HU for Onyx vs. 7.56 ± 1.34 HU for PHIL in CT (*p* < 0.001) and 41.88 ± 7.22 density units (DU) for Squid vs. 35.22 ± 5.84 DU for PHIL in CBCT (*p* = 0.044). Qualitative analysis showed less artifacts for PHIL compared to Onyx and Squid in both imaging modalities while there was no difference between Onyx and Squid regarding the definition of the nidus (*p* > 0.999).

**Conclusion:**

In this novel three-dimensional in vitro AVM model, IAs were higher for the EVOH/tantalum-based LEAs Onyx and Squid compared to iodine-based PHIL. Onyx induced the highest degree of IAs with only minor differences to Squid.

## Introduction

Besides conservative management, treatment options for cerebral arteriovenous malformations (AVMs) include endovascular embolization, microneurosurgery and radiosurgery [1, 2]. Especially endovascular embolization using liquid embolic agents (LEAs) is an increasingly applied treatment option [3, 4]. Over the past years the range of commercially available LEAs has increased steadily. One of the most commonly used LEAs is Onyx (Medtronic, Irvine, CA, USA), a nonadhesive agent consisting of ethylene vinyl alcohol (EVOH) copolymer, dimethyl-sulfoxide (DMSO) and tantalum powder [5–7]. Another nonadhesive LEA is Squid (Balt, Montmorency, France), which became available in 2012 [8]. Both products are based on the same chemical substances, while the main difference between Onyx and Squid is the grain size of the micronized tantalum powder. Squid features a smaller grain size aiming to enhance the homogeneity in radiopacity and improving the visibility during longer injections times.

Besides these two EVOH-based LEAs, a further available and recently introduced LEA is the precipitating hydrophobic injectable liquid (PHIL; MicroVention, Aliso Viejo, CA, USA) [9]. Unlike the EVOH-based LEAs, PHIL is composed of two specific copolymers (polylactide-co-glycolide and polyhydroxyethylmethacrylate) which are covalently bound to triiodophenol as its radiopaque component.

An often reported drawback of these commonly used non-adhesive LEAs is the generation of imaging artifacts in conventional computed tomography (CT) and cone-beam CT (CBCT) [10–12]. Since cerebral AVMs have an increased risk of periprocedural and postprocedural hemorrhage, LEA-related artifacts may represent a crucial obstacle for detection of intracranial blood during or after embolization in both of the aforementioned medical imaging modalities [13]. Furthermore, especially complex intracranial AVMs may not be completely occluded by endovascular embolization, requiring subsequent radiation therapy afterwards [1]. The recordings for the planning of the corresponding radiation treatment are usually based on conventional CT imaging [14]. In this context, embolization-related imaging artifacts may represent a crucial obstacle for an adequate and safe treatment planning [15–17].

So far there are only a few clinical and preclinical reports available, which investigated the imaging artifacts of LEAs in conventional CT or CBCT [10–12, 18]. To our knowledge an experimental AVM model has been applied in only one of these studies, assessing and comparing the imaging artifacts of Onyx and PHIL [10]. The aim of the present study was the investigation of imaging artifacts of the most commonly used non-adhesive LEAs Onyx 18, Squid 18 and PHIL 25% in conventional CT and CBCT in a novel three-dimensional in vitro AVM model.

## Material and Methods

### Preparation of the Experimental AVM Model

**Fig. 1:**
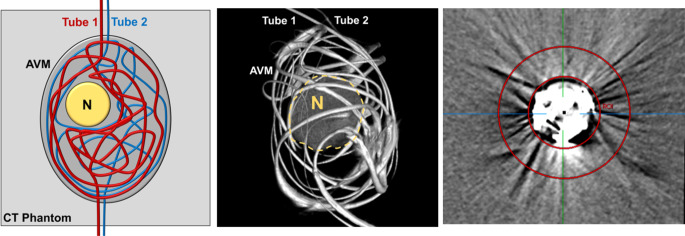
Schematic illustration and volume rendering of an example of a three-dimensional AVM model. Two DMSO-compatible tubes with different diameters were irregularly configured around an artificial nidus (*N*), inserted into an ovate plastic container and filled with embolic agent, resembling a partially embolized AVM. The AVM model was placed within a CT phantom for image acquisition. The right image shows the customized feature of the MITK software which allowed us to place a defined region of interest (ROI) with a donut-shaped configuration adjacent to and surrounding the experimental AVM models for quantitative image analysis. The AVM model in the picture was filled with Squid 18 and a standard brain window with a width of 80 HU and a length of 40 HU was applied

A schematic description of the experimental AVM model is illustrated in Fig. 1. Two different sizes of DMSO-compatible tubes, each with a length of 600 mm, were manually configured as an AVM and inserted into a thin-walled plastic container. Before preparation, different DMSO-compatible tubes were tested while the tubes with less radiopacity were used. The ovate plastic container had a diameter of 32 mm and a length of 45 mm. Tube 1 had an inner diameter of 1.6 mm and an outer diameter of 3.2 mm, while tube 2 had an inner diameter of 1.0 mm and an outer diameter of 1.8 mm. A manually configured artificial nonembolized AVM nidus, consisting of natural rubber latex, with a total volume of 2 ml, filled with saline (NaCl 0.9%) and CT contrast medium (Imeron 300, Bracco Imaging Deutschland GmbH, Konstanz, Germany) in a ratio of 1:4, similar to Tan et al. [19], was placed at its center.

Initially all tubes were flushed with warm saline (38.0 °C, NaCl 0.9%) while there was no continuous flush during the filling procedure. All LEAs were prepared in accordance with the manufacturer’s instructions and as recommended for clinical use. In a next step, complete filling of the experimental AVM models was performed with Onyx 18, Squid 18 and PHIL 25%. Therefore, both tubes were filled simultaneously by manual pulsatile injection using 1 ml, DMSO-compatible syringes. The filling procedure was performed with an average flow of 1 ml per 5 min. A total volume of 1.20 ml was injected into tube 1 while 0.47 ml were injected into tube 2. Ten saline-filled AVM models were investigated as well, serving as a control group.

### Imaging

Image acquisition for conventional CT and CBCT was performed with standard settings according to clinical routine. Conventional CT imaging was performed on a 64-slice multidetector, single source scanner (Somatom Definition AS, Siemens Healthineers, Erlangen, Germany) with a tube voltage of 120 kV and a tube current of 20 mAs. Reconstruction of the conventional CT images was conducted with a J40s kernel. CBCT was obtained on a biplanar angiography system (Artis Q, Siemens Healthineers) with the following parameters: 20 s rotational acquisition generating 500 projections with an angular step of 0.4 ° for a total coverage of 200 ° with a pulse length of 12.5 ms, a tube voltage of 109 kV and a dose per frame of 1.82 μGy. Both conventional CT and CBCT images were reconstructed with a slice thickness of 4 mm in the axial plane.

**Fig. 2:**
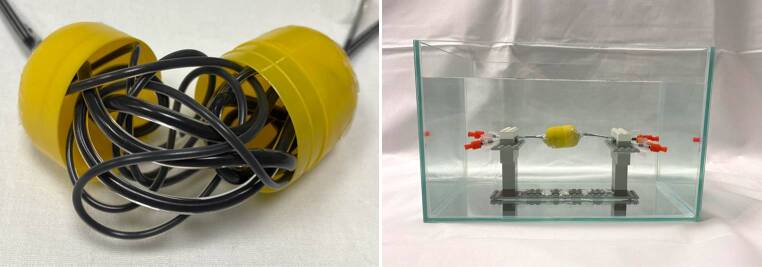
Illustration of the experimental setup of the custom-made AVM model and CT phantom. Each of the filled AVM models was inserted into the illustrated custom-made CT phantom for conventional CT and cone-beam CT image acquisition. Therefore, the container was filled with saline and contrast medium with an average density similar to brain tissue. The plastic fixture inside the imaging phantom allowed us to hang the AVM models for an optimal measurement of the surrounding imaging artifacts. The illustrated image shows an AVM model filled with Onyx 18

All filled AVM models were inserted into a custom-made CT phantom consisting of saline and contrast medium with an average density similar to brain tissue (conventional CT: mean: 33.906 HU; standard deviation [SD]: 0.397 HU), following the model of Daubner et al. [20]. Prior to the image acquisition it was ensured that the interior of the plastic container and thus all structures of the AVM model were fully surrounded by fluid of the CT phantom. To guarantee an optimal spread of the artifacts, the beam path for all scans was orthogonal to the filled AVM models. An illustration of the experimental set-up of the custom-made CT phantom and the AVM models is demonstrated in Fig. 2.

### Quantitative Image Analysis

Quantitative analysis of the LEA-related imaging artifacts was performed using the Medical Imaging Interaction Toolkit (MITK; German Cancer Research Center (DKFZ), Heidelberg, Germany) [21]. As described previously [12], a customized feature of the MITK software allowed us to place a defined region of interest (ROI) with a donut-shaped configuration adjacent to and surrounding the experimental AVM models in the axial plane. For quantitative analysis, the customized ROI was placed on the central image slice of the upper third, the middle third and the lower third, so that imaging artifacts of different positions were taken into account. For both the conventional CT and the CBCT images the inner diameter of the donut-shaped ROI was set at 36 mm and the outer diameter at 66 mm while the experimental AVM models had a maximum diameter of 32 mm. Window width and level was adjusted manually to ensure an adequate placement of the ROI in each position.

Since streak artifacts in conventional CT and CBCT usually consist of areas of high density and low density, the degree of imaging artifacts was assessed by calculating the SD of the HU for conventional CT and the SD of the density units (DU) for CBCT in each ROI [17]. This procedure has the advantage that the imaging artifacts might not be cancelled out as might be the case for the mean density values.

### Qualitative Image Analysis

Qualitative analysis of the conventional CT and CBCT images was performed by two different readers (reader 1 with 4 years; reader 2 with 7 years experience in diagnostic imaging) on a picture archiving and communication system workstation (CENTRICITY PACS 4.0; GE Healthcare, Barrington, IL, USA). To improve the quality of the analysis, a second analysis was performed by each reader after 3 months. Each reader was blinded to the type of LEA. To ensure an adequate evaluation of the artificial nidus, similar to clinical practice, a standard CT angiography window with a width of 1000 HU and a level of 200 HU was chosen for conventional CT images [22]. Since there is no standardized angiography window for CBCT so far, window width was set at 250 DU while the window level was set at 90 DU, similar to Struffert et al. [23]. The observers were not allowed to adjust the window.

The feasibility of defining the artificial nidus within the center of each AVM using the defined CT and CBCT windows was graded by a five-point scale [24]: (1) major artifacts, artificial nidus not definable, (2) marked artifacts, artificial nidus partially definable, (3) moderate artifacts, artificial nidus moderately definable, (4) minor artifacts, artificial nidus easily definable and (5) no artifacts, artificial nidus totally definable.

### Statistics

GraphPad Prism software (version 8.4.3, La Jolla, CA, USA) was used for statistical analysis. The interreader and intrareader agreement for qualitative image analysis was assessed by using the Cohen’s κ coefficient [25]. The κ values were interpreted as follows: $\leq$0.20 no agreement, 0.21–0.39 minimal agreement, 0.40–0.59 weak agreement, 0.60–0.79 moderate agreement, 0.80–0.90 strong agreement and ≥ 0.90 almost perfect agreement [26]. Kruskal-Wallis test was performed to evaluate statistical differences between the study groups. The data of the quantitative analysis is presented as mean ± SD. Dunn’s test for multiple comparisons using statistical hypothesis testing was performed as a post hoc test, to evaluate the differences between the individual study groups. The significance level was defined at *P* < 0.05.

## Results

**Fig. 3:**
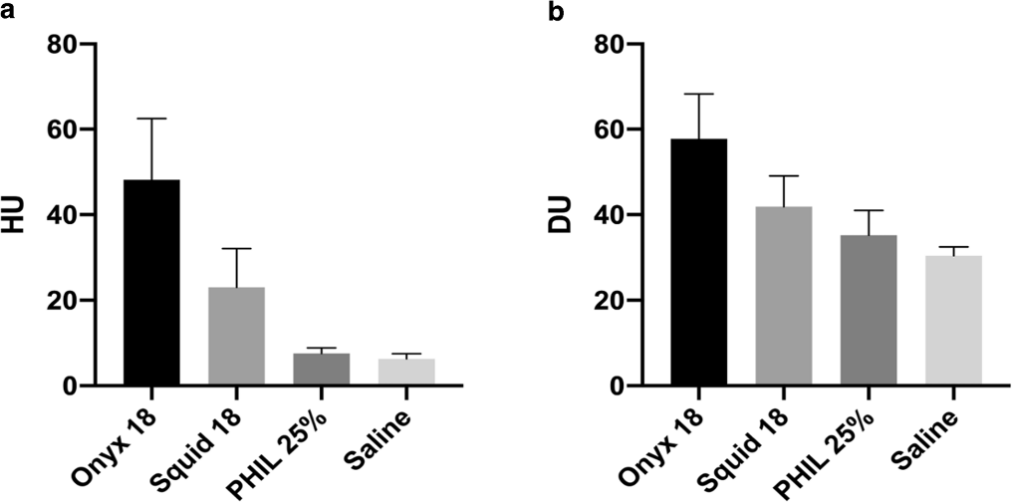
Illustration of the results of the quantitative image analysis. A different degree of artifacts was observed in the standardized ROI between all four study groups in conventional CT (**a**) and cone-beam CT (**b**). Kruskal-Wallis test showed significant differences between all groups, except for PHIL 25% vs. saline in conventional CT. For both imaging modalities Onyx 18 produced the highest degree of artifacts. *Bars* mean; *whiskers* standard deviation, *HU* Hounsfield units, *DU* density units

The results of the quantitative image analysis are summarized in Fig. 3, and in Tables 1 and 2.

**Table 1:** Summary of the results of the quantitative (A) and qualitative (B) imaging analyses

**(A) LEA**	**Mean HU** ± **SD of the ROI in conventional CT**	**Mean DU** ± **SD of the ROI in cone-beam CT**	***p*-value**^**a**^
**Onyx 18**	48.15 ± 14.32 HU	57.77 ± 10.54 DU	*p* < 0.001
**Squid 18**	22.94 ± 9.12 HU	41.88 ± 7.22 DU	
**PHIL 25%**	7.56 ± 1.34 HU	35.22 ± 5.84 DU	
**Saline**	6.33 ± 1.21 HU	30.44 ± 2.07 DU	
**(B) LEA**	**Mean** ± **SD of the qualitative analysis in conventional CT**	**Mean** ± **SD of the qualitative analysis in cone-beam CT**	***p*-value**^**a**^
**Onyx 18**	2.43 ± 0.44	1.78 ± 0.38	*p* < 0.001
**Squid 18**	2.70 ± 0.44	1.90 ± 0.53	
**PHIL 25%**	3.88 ± 0.56	2.78 ± 0.41	
**Saline**	5.00 ± 0.00	4.60 ± 0.48	

^a^Kruskal-Wallis test; for the *p*-values of the post hoc test, see Table 2

*LEA* liquid embolic agent, *HU* Hounsfield units, *DU* density units, *SD* standard deviation, *ROI* region of interest

**Table 2:** Summary of the results of the post hoc Dunn’s test

**(A) LEA in conventional CT**	**Saline**	**PHIL 25%**	**Squid 18**	**(B) LEA in** **cone-beam CT**	**Saline**	**PHIL 25%**	**Squid 18**
**Onyx 18**	*p* *<* *0.001*	*p* *<* *0.001*	*p* *=* *0.019*	**Onyx 18**	*p* *<* *0.001*	*p* *<* *0.001*	*p* *=* *0.005*
**Squid 18**	*p* *<* *0.001*	*p* *<* *0.001*	–	**Squid 18**	*p* *<* *0.001*	*p* *=* *0.044*	–
**PHIL 25%**	*p* = 0.502	–	–	**PHIL 25%**	*p* *=* *0.04*	–	–
**(C) LEA in conventional CT**	**Saline**	**PHIL 25%**	**Squid 18**	**(D) LEA in** **cone-beam CT**	**Saline**	**PHIL 25%**	**Squid 18**
**Onyx 18**	*p* *<* *0.001*	*p* *<* *0.001*	*p* > 0.999	**Onyx 18**	*p* *<* *0.001*	*p* *=* *0.001*	*p* > 0.999
**Squid 18**	*p* *<* *0.001*	*P* *=* *0.004*	–	**Squid 18**	*p* *<* *0.001*	*p* *=* *0.011*	–
**PHIL 25%**	*p* *=* *0.032*	–	–	**PHIL 25%**	*p* *=* *0.005*	–	–

*P*‑values of the quantitative analyses for conventional CT (A) and CBCT (B). Significant difference was observed between all study groups, except for PHIL 25% vs. saline in conventional CT

*P*‑values of the qualitative analyses for conventional CT (C) and CBCT (D). There was no statistically significant difference between the EVOH-based LEAs Onyx 18 and Squid 18 while all other groups demonstrated a significantly different degree of artifacts in both imaging modalities

Italic type indicates statistical significance

*LEA* liquid embolic agent

Kruskal-Wallis tests showed a difference (*p* < 0.001) in the degree of artifacts between all study groups for conventional CT and CBCT. For both imaging modalities, Onyx 18 induced a higher degree of artifacts compared to Squid 18 and PHIL 25% (CT: *p* = 0.019 for Squid and *p* < 0.001 for PHIL; CBCT: *p* = 0.005 for Squid and *p* < 0.001 for PHIL). Furthermore, a higher degree of imaging artifacts caused by Squid 18 compared to PHIL 25% was observed for conventional CT as well as for CBCT (CT: *p* < 0.001; CBCT: *p* = 0.044). The intensity of imaging artifacts was lowest for the saline-filled AVM models (control group), reaching statistical significance in all groups for both imaging modalities, except for PHIL 25% in conventional CT (*p* = 0.502).

**Fig. 4:**
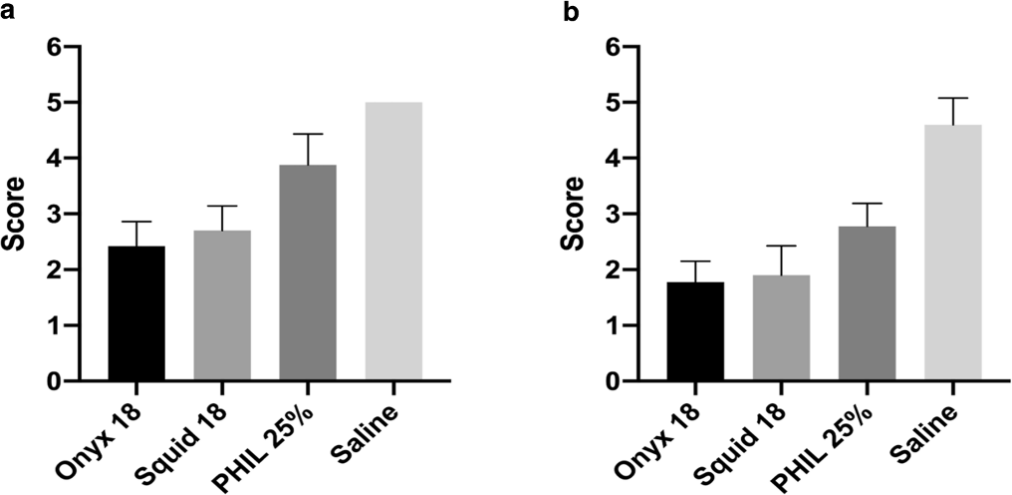
Illustration of the results of the qualitative image analysis. Qualitative analysis showed a different degree of LEA-related imaging artifacts for conventional CT (**a**) and cone-beam CT (**b**). The definition of the nidus by a five-point scale was more precise within the AVM models which were filled with PHIL 25% compared to the EVOH-based LEAs Onyx 18 and Squid 18. There was no difference between the EVOH-based LEAs Onyx 18 and Squid 18 in both imaging modalities. *Bars* mean; *whiskers* standard deviation

The results of the qualitative image analysis are summarized in Fig. 4, and in Tables 1 and 2.

**Fig. 5:**
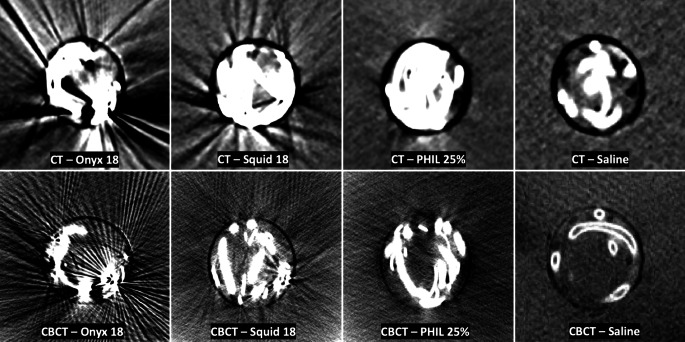
Representative CT and CBCT images of the novel AVM model in axial plane. For conventional CT a standard brain window with a width of 80 HU and a length of 40 HU and for CBCT a width of 350 DU and a length of 30 DU was set. A higher degree of imaging artifacts for the EVOH-based LEAs (Onyx and Squid) was observed compared to PHIL which uses iodine as its radiopaque component

Interreader and intrareader reliability showed a strong agreement for conventional CT (interreader: κ = 0.865; range: 0.776–0.954/intrareader: κ = 0.881; range: 0.798–0.965) as well as for CBCT (interreader: κ = 0.863; range: 0.776–0.953/intrareader: κ = 0.829; range: 0.729–0.928) for the definition of the artificial nidus at the center of each AVM. In both imaging modalities, the definition of the nidus was more precise within the AVM models filled with PHIL 25% (CT: 3.88 ± 0.56; CBCT: 2.78 ± 0.41) compared to the EVOH-based LEAs Onyx 18 (CT: 2.43 ± 0.44, *p* < 0.001; CBCT: 1.78 ± 0.38, *p* = 0.001) and Squid 18 (CT: 2.70 ± 0.44, *p* = 0.004; CBCT: 1.90 ± 0.53, *p* = 0.011). There was no difference between Onyx 18 and Squid 18 in conventional CT (*p* > 0.999) and CBCT (*p* > 0.999) regarding the qualitative definition of the artificial nidus within the AVM models. Level of significance was reached for all LEAs compared to the saline filled control groups in conventional CT and CBCT. Representative CT and CBCT images of the examined LEAs are demonstrated in Fig. 5.

## Discussion

Besides microneurosurgery and radiation therapy, endovascular embolization using LEAs is an established treatment option for intracranial vascular malformations such as AVMs and DAVFs [1, 2]. As described initially, LEA-related imaging artifacts may represent a crucial obstacle for the detection of periprocedural intracranial hemorrhage or for an adequate treatment planning of subsequent radiation therapy [14–17]. In this study we tested a newly developed three-dimensional in vitro AVM model, resembling a partially embolized AVM, and demonstrated that the iodine-based LEA PHIL produces less imaging artifacts compared to the tantalum-based LEAs Onyx and Squid in CT and CBCT. 

So far there are only a limited number of clinical and preclinical studies available, analyzing and comparing the degree of imaging artifacts of LEAs [10–12]. An experimental AVM model has been applied for only one of these studies. Using this model, Vollherbst et al. demonstrated a higher degree of embolization-related imaging artifacts for Onyx 18 compared to PHIL 25% in conventional CT as well as CBCT in an animal model using the porcine rete mirabile as an AVM model [10]. The other available studies used an experimental two-dimensional in vitro tube model for investigation [11, 12]. Schmitt et al. demonstrated a difference in the degree of artifacts between the EVOH-based LEAs compared to PHIL while both studies achieved different results regarding the imaging artifacts of Onyx compared to Squid [11, 12]. The utilization of a more complex three-dimensional AVM model for investigation of the LEA-related artifacts is one major advantage of the present work. The simple experimental set-up of the tube model benefits from a higher level of reproducibility and comparability, while the present three-dimensional model much better resembles an intracranial vascular malformation. Compared with the rete mirabile, however, which can show variations depending on different factors, such as the size, weight or race of the animals, the AVM model which was used in this study features a high level of standardization regarding length and diameter of the DMSO-compatible tubes as well as the size and configuration of the artificial nidus. Despite these advantages, especially in terms of morphology and flow dynamics, the present model still features distinct differences from a human AVM, while there are only limited options for such models due to the required DMSO-compatibility. In the abovementioned preclinical studies, imaging artifacts were analyzed using various manually positioned ROIs in the area of imaging artifacts [10, 11]. This method can bear drawbacks, such as unintended placement of the ROI in areas of high or low artifacts as well as averaging of artifacts in the manually positioned ROI. Furthermore, there was a relatively low number of cases in these studies, while in the present work 10 experiments per study group were performed and a customized donut-shaped ROI was used for the assessment of the artifacts. This customized feature of the MITK software allowed us to set a standardized donut-shaped ROI adjacent to and surrounding each filled AVM model with the possibility of considering all LEA-induced artifacts and at the same time not taking the filled AVM models into account. To ensure a more adequate analysis by considering high and low vascularized regions as well as the artificial nidus, quantitative analysis was performed at 3 different levels. Nevertheless, the different configuration of the filled tubes within each plastic container might introduce certain bias in artifact generation. Additionally, investigation of ten saline filled AVM models was conducted, serving as a control group.

For the first time, the imaging artifacts of Squid 18 have been assessed in an AVM model in conventional CT and CBCT. Our findings can mainly be attributed to the admixed chemical elements which cause radiopacity [17]. The higher atomic number of tantalum (atomic number 73) as part of Onyx 18 and Squid 18 compared to iodine (atomic number 53) as part of PHIL 25% seems to have a major impact on the generation of imaging artifacts. Furthermore, there was a quantitatively higher degree of artifacts in conventional CT and CBCT for Onyx 18 compared to Squid 18. Since both LEAs contain similar quantities of EVOH (6%), DMSO and tantalum powder, there is a smaller size of the tantalum particles in Squid 18. This smaller grain may lead to an improved distribution of the tantalum within a defined volume and thus resulting in less X‑ray diffraction and less imaging artifacts. Another reason might be the prolonged suspension time of the smaller tantalum particles in Squid 18. As recently described by Mason et al., sedimentation in Onyx 18 is nearly three times faster compared to Squid 18 [27]. According to these findings, faster deposition may lead to areas of concentrated tantalum powder and thus resulting in an increased degree of imaging artifacts. In order to prevent additional influence because of different deposition times, image acquisition of the present study was performed immediately after filling. Despite Onyx 18 and Squid 18 producing a different degree of artifacts in quantitative analysis, there was no statistical difference between them in qualitative analysis, which can be explained by the high degree of artifacts of both of these agents. Both EVOH-based LEAs caused more imaging artifacts compared to PHIL 25%. It seems that in the predefined viewing window no adequate differentiation of the artificial nidus might be possible after exceeding a determined level of artifacts and that this threshold is surpassed to such an extent that in qualitative analysis significant differences between both could no longer be observed. The option of a manual window adjustment may have had an impact on these findings but the aim of the present qualitative analysis was to enable a standardized comparability between the individual LEAs. In our study, the results of the qualitative analysis revealed similar findings for both imaging modalities, after which no difference in the degree of imaging artifacts between Onyx 18 and Squid 18 could be observed.

As indicated in the introduction, the major negative impacts of the imaging artifacts produced by LEAs in clinical practice is that they can impede the detection of periprocedural intracranial hemorrhage or the planning of a subsequent stereotactic radiosurgery [13–17]. Postprocessing techniques could potentially improve these drawbacks, for example iterative metal artifact reduction algorithms or gated multidetector CT [18, 28]. A further approach to improve the planning and performance of a stereotactic radiosurgical treatment is to apply additional digital subtraction angiography or magnetic resonance imaging [14, 29].

We acknowledge that this study has several limitations. First, besides the highly standardized analysis and comparison of the LEA-related imaging artifacts, the transferability of the novel in vitro model to clinical practice still has limitations. Second, only two different sizes of tubes were used to build the AVM models, while the vessel size in human vascular malformations is much more variable. Furthermore, the used tubes are slightly radiopaque, which might modify the degree of artifacts caused by the embolic agents. The AVM models were not flushed continuously with saline during the LEA injection which may have a separate impact on imaging artifacts.

## Conclusion

The novel three-dimensional in vitro AVM model is feasible for the investigation of imaging artifacts of LEAs. The degree of imaging artifacts of the most commonly used LEAs in this model differed significantly. In conventional CT and CBCT artifacts were higher for the EVOH/tantalum-based LEAs Onyx 18 and Squid 18 compared to PHIL 25% which uses iodine as its radiopaque component. Onyx 18 induced the highest degree of artifacts while only minor differences in comparison to Squid 18 could be observed.

## Abbreviations

AVM: Arteriovenous malformation
CBCT: Cone-beam computed tomography
EVOH: Ethylene vinyl alcohol
LEA: Liquid embolic agent
